# Type I collagen promotes triple-negative breast cancer progression through a UBE2V1–ACSL5 regulatory axis

**DOI:** 10.3389/fonc.2026.1916886

**Published:** 2026-09-03

**Authors:** Lu Liu, Yida Wang, Haiyue You, Cenzhu Wang, Xinfeng Yang, Feng Zhang, Danping Wu, Xin Ning, Zhiwen Qian, Ying Jiang, Yan Zhang, Xiaoqian Zhao, Tingting Han

**Affiliations:** 1 Department of Oncology, Wuxi Maternity and Child Health Care Hospital, Women’s Hospital of Jiangnan University, Jiangnan University, Wuxi, China; 2 Department of Oncology, Wuxi Maternal and Child Health Care Hospital, Wuxi Medical Center, Nanjing Medical University, Wuxi, China; 3 Department of Oncology, The Affiliated Wuxi People’s Hospital of Nanjing Medical University, Wuxi People’s Hospital, Wuxi Medical Center, Nanjing Medical University, Wuxi, China

**Keywords:** ACSL5, extracellular matrix, protein stability, triple-negative breast cancer, type I collagen, UBE2V1, ubiquitination

## Abstract

**Introduction:**

Triple-negative breast cancer (TNBC) is characterized by aggressive behavior and extensive extracellular-matrix remodeling. Type I collagen (COL I), a major stromal component, promotes tumor progression, but the downstream tumor-cell mechanisms remain incompletely defined. We investigated whether COL I regulates acyl-CoA synthetase long-chain family member 5 (ACSL5) through ubiquitin-conjugating enzyme E2 variant 1 (UBE2V1) and whether this relationship contributes to malignant behavior.

**Methods:**

We integrated public-dataset analyses, transcriptome sequencing, and in vitro functional and biochemical assays. MDA-MB-231 and BT-549 cells were exposed to COL I, with ACSL5 overexpression or UBE2V1 knockdown as indicated. ACSL5 expression and stability were assessed by qPCR, western blotting, CHX-chase analysis, CQ or MG132 treatment, ubiquitination assays, immunoprecipitation-mass spectrometry, and co-immunoprecipitation.

**Results:**

COL I enhanced proliferation, colony formation, migration, and invasion and reduced ACSL5 protein abundance in both TNBC cell lines. ACSL5 overexpression partially reversed these malignant phenotypes. COL I did not significantly alter ACSL5 mRNA expression by qPCR but accelerated ACSL5 protein loss; MG132, but not chloroquine, partially restored ACSL5, and total ACSL5 ubiquitination increased after COL I treatment. Immunoprecipitation-mass spectrometry and co-immunoprecipitation identified UBE2V1 as an ACSL5-associated protein whose expression and association with ACSL5 were enhanced by COL I. UBE2V1 knockdown partially restored ACSL5 protein expression and significantly attenuated COL I-induced migration and invasion. Public-dataset analyses further showed reduced ACSL5 expression in breast cancer and associations between ACSL5 and immune-infiltration features; these observations were exploratory and were not supported by direct immune-functional assays.

**Discussion:**

These findings identify a COL I-UBE2V1-ACSL5 regulatory relationship that contributes to TNBC malignant progression. The data support UBE2V1-associated regulation of proteasome-sensitive ACSL5 loss, while the complete ubiquitination machinery and the in vivo, clinical, and immune relevance remain to be defined.

## Introduction

Breast cancer is a leading malignancy among women worldwide (1). Triple-negative breast cancer (TNBC) represents a clinically challenging molecular subtype defined by the absence of estrogen receptor (ER), progesterone receptor (PR), and human epidermal growth factor receptor 2 (HER2) expression (2–4). Because TNBC does not benefit from endocrine therapy or HER2-targeted therapy, systemic treatment has historically relied mainly on chemotherapy, with immunotherapy emerging as an important but still incompletely effective strategy (5, 6). Compared with other breast cancer subtypes, TNBC is characterized by greater aggressiveness, a higher risk of recurrence and metastasis, and poorer clinical prognosis (3, 7). Although immune checkpoint blockade (ICB) has provided new therapeutic opportunities for selected patients with TNBC, the overall response rate remains limited, indicating the presence of complex immune escape mechanisms (6, 8–10).

The tumor microenvironment (TME) is a critical determinant of TNBC progression and therapeutic response (11, 12). The extracellular matrix (ECM), a major component of the TME, provides not only physical support but also biochemical and biomechanical signals that regulate cell signaling, metabolic reprogramming, immune-cell infiltration, and therapeutic resistance (12, 13). Type I collagen (COL I) is one of the most abundant collagen types within the ECM (14, 15). Excessive COL I deposition increases matrix stiffness, restricts immune-cell penetration into tumor parenchyma, and promotes tumor-cell proliferation, migration, invasion, metastasis, and treatment resistance (16–18). Therefore, clarifying the downstream mechanisms by which COL I reshapes TNBC biology is important for understanding stromal regulation of tumor-cell behavior and therapeutic response.

Acyl-CoA synthetase long-chain family member 5 (ACSL5) is a member of the long-chain acyl-CoA synthetase (ACSL) family and participates in fatty-acid activation and lipid metabolic regulation (19–21). Beyond its canonical metabolic functions, ACSL5 has recently been implicated in tumor antigen presentation, immune infiltration, and sensitivity to antitumor immunity (21, 22). Existing evidence suggests that ACSL5 may regulate major histocompatibility complex class I (MHC-I)-related antigen presentation and thereby influence tumor immunogenicity (22). However, the upstream mechanisms controlling ACSL5 expression and protein stability in TNBC remain unclear.

UBE2V1, also known as UEV1A, is a ubiquitin-conjugating E2 enzyme variant involved in ubiquitination-related signaling and protein homeostasis (23–25). Unlike catalytically active E2 enzymes, UBE2V1 lacks the canonical active-site cysteine and generally functions with a catalytic E2 partner, such as UBE2N/UBC13, rather than independently catalyzing ubiquitin transfer (23–25). UBE2V1 has been linked to tumor progression, metastasis, and immune-related signaling in several cancers (26–29). Nevertheless, whether UBE2V1 is involved in COL I-mediated regulation of ACSL5 protein stability has not been clearly established.

In the present study, we integrated bioinformatics analyses, transcriptome screening, and *in vitro* functional and biochemical assays to investigate the regulatory relationship among COL I, UBE2V1, and ACSL5. We examined whether COL I alters ACSL5 protein stability, whether UBE2V1 is associated with this regulation, and whether UBE2V1 is required for COL I-induced ACSL5 downregulation and invasive behavior. Immune-related public-dataset analyses were retained as hypothesis-generating observations rather than direct evidence of immune escape.

## Materials and methods

### Bioinformatics analyses

Public transcriptomic and clinical data were obtained from The Cancer Genome Atlas Breast Invasive Carcinoma cohort (TCGA-BRCA). Gene-expression values were transformed as log2(TPM + 1) before downstream analysis. A collagen-formation score was calculated for each tumor sample by single-sample gene set enrichment analysis (ssGSEA) using the REACTOME_COLLAGEN_FORMATION gene set from the Molecular Signatures Database (MSigDB; C2:CP: REACTOME; Reactome pathway R-HSA-1474290). Tumors were categorized into collagen-high and collagen-low groups according to the median collagen-formation score. StromalScore and ESTIMATEScore were calculated using the ESTIMATE algorithm. Differences between the collagen-high and collagen-low groups were assessed using a two-sided Wilcoxon rank-sum test. Gene Ontology enrichment analysis was performed using the “clusterProfiler” package, and P values were adjusted for multiple comparisons using the Benjamini–Hochberg method. An adjusted P value < 0.05 was considered statistically significant.

### Cell culture and treatment

Human TNBC cell lines MDA-MB-231 and BT-549 were maintained in the corresponding complete culture media at 37 °C in a humidified incubator containing 5% CO2. Type I collagen (COL I; Corning Collagen I, Rat Tail, 354236) was diluted in complete culture medium and added directly to the cells at a final concentration of 50 μg/mL for 24 h. Control cells received an equal volume of the corresponding vehicle. The treatment concentration and duration were selected according to preliminary optimization and previously reported collagen-treatment conditions in breast cancer cells. According to the experimental design, cells were transfected with ACSL5 overexpression plasmids or UBE2V1-targeting siRNA, or treated with cycloheximide, MG132, or chloroquine. Unless otherwise indicated, all quantitative cell experiments were performed using three independent biological replicates.

### UBE2V1 knockdown

Small interfering RNA targeting human UBE2V1 and a matched scrambled negative-control siRNA were synthesized by KEYGEN China. The sense sequence of siUBE2V1 was 5′-GGACAGUGUUACAGCAAUU-3′. MDA-MB-231 and BT-549 cells were seeded one day before transfection and transfected at approximately 50–60% confluence using Lipofectamine RNAiMAX (Thermo Fisher Scientific) according to the manufacturer’s instructions. The final siRNA concentration was 10 nM. At 48 h after transfection, cells were treated with or without COL I for an additional 24 h. The four experimental groups were siNC, siNC + COL I, siUBE2V1, and siUBE2V1 + COL I. UBE2V1 knockdown efficiency and ACSL5 protein expression were evaluated by western blotting.

### Transcriptome sequencing and candidate-molecule screening

To identify downstream molecules regulated by COL I in TNBC cells, transcriptome sequencing was performed in COL I-treated and control TNBC cells. Differentially expressed genes were screened and functionally annotated, with particular attention to genes associated with lipid metabolism, cell proliferation, tumor invasion, antigen presentation, and immune regulation. ACSL5 was selected for downstream validation after integrating its expression change with functional annotation and immune-related bioinformatics evidence.

### Plasmid transfection

Cells were transfected at approximately 70% confluence. ACSL5 overexpression plasmids and matched negative-control plasmids were introduced using Lipofectamine 3000 following the manufacturer’s protocol. Cells were then cultured for 24–96 h, depending on the downstream assay, and the efficiency of ACSL5 overexpression was checked by western blotting.

### Western blotting and qPCR

After the indicated treatments, total protein and total RNA were extracted from TNBC cells. Protein concentration was measured using a bicinchoninic acid (BCA) assay. Equal amounts of protein were resolved by sodium dodecyl sulfate-polyacrylamide gel electrophoresis (SDS-PAGE), transferred to polyvinylidene fluoride (PVDF) membranes, blocked, incubated with primary and secondary antibodies, and detected by chemiluminescence. For quantitative polymerase chain reaction (qPCR), total RNA was reverse-transcribed into complementary DNA (cDNA), and target-gene expression was measured with a SYBR Green-based qPCR system. Relative expression was calculated after normalization to the internal reference gene.

### Cell counting kit-8, colony-formation, migration, and invasion assays

The CCK-8 assay was used to assess short-term cell proliferation. Treated cells were seeded into 96-well plates, and CCK-8 reagent was added at 24, 48, and 72 h. Absorbance at 450 nm was measured using a microplate reader. Colony-formation assays were performed to evaluate long-term proliferative capacity. Treated cells were seeded at low density in six-well plates, cultured for 7–14 days, fixed, stained with crystal violet, and counted. Transwell assays were used to evaluate cell migration and invasion. For migration assays, cells were seeded into uncoated Transwell chambers; for invasion assays, chambers were pre-coated with Matrigel or an equivalent basement membrane matrix. After 24–48 h, migrated or invaded cells were fixed, stained, imaged, and quantified.

### CHX chase, protein-degradation pathway inhibition, and ubiquitination assay

ACSL5 protein stability was evaluated by CHX chase analysis. CHX was added to control and COL I-treated cells, and protein lysates were collected at 0, 3, 6, 9, and 12 h for western blotting. ACSL5 degradation kinetics were examined by western blotting. To determine the degradation pathway, CQ was used to inhibit the autophagy-lysosome pathway, whereas MG132 was used to inhibit the ubiquitin-proteasome system (UPS). ACSL5 protein levels were then detected by western blotting. To examine ACSL5 ubiquitination, cells were pretreated with MG132, followed by immunoprecipitation (IP) to enrich ACSL5 protein. Ubiquitinated ACSL5 was detected by western blotting using an anti-ubiquitin antibody.

### Immunoprecipitation, co-immunoprecipitation, and mass spectrometry

IP was performed to enrich ACSL5 and its interacting protein complexes, followed by mass spectrometry (MS) to screen potential interacting proteins. Candidate ubiquitination-related molecules were identified by integrating MS results with UPS-related annotation, transcriptome data, and bioinformatics screening. UBE2V1 was selected as a candidate mediator. Co-immunoprecipitation (Co-IP) was subsequently performed to validate the interaction between UBE2V1 and ACSL5 and to compare changes in UBE2V1-ACSL5 interaction strength before and after COL I treatment.

### Statistical analysis

Statistical analyses were conducted in GraphPad Prism9.5.0. Unless otherwise stated, data are shown as mean ± standard deviation (SD). Two-group comparisons were made using Student’s t-test. Comparisons among multiple groups were performed using one-way analysis of variance (ANOVA), followed by Tukey’s multiple-comparison test. Experiments involving both time and treatment factors were analyzed by two-way ANOVA followed by Tukey’s multiple-comparison test. All tests were two-sided, and *P* < 0.05 was considered statistically significant.

## Results

### Collagen-related transcriptomic signatures are associated with increased stromal features in breast cancer

**Figure 1:**
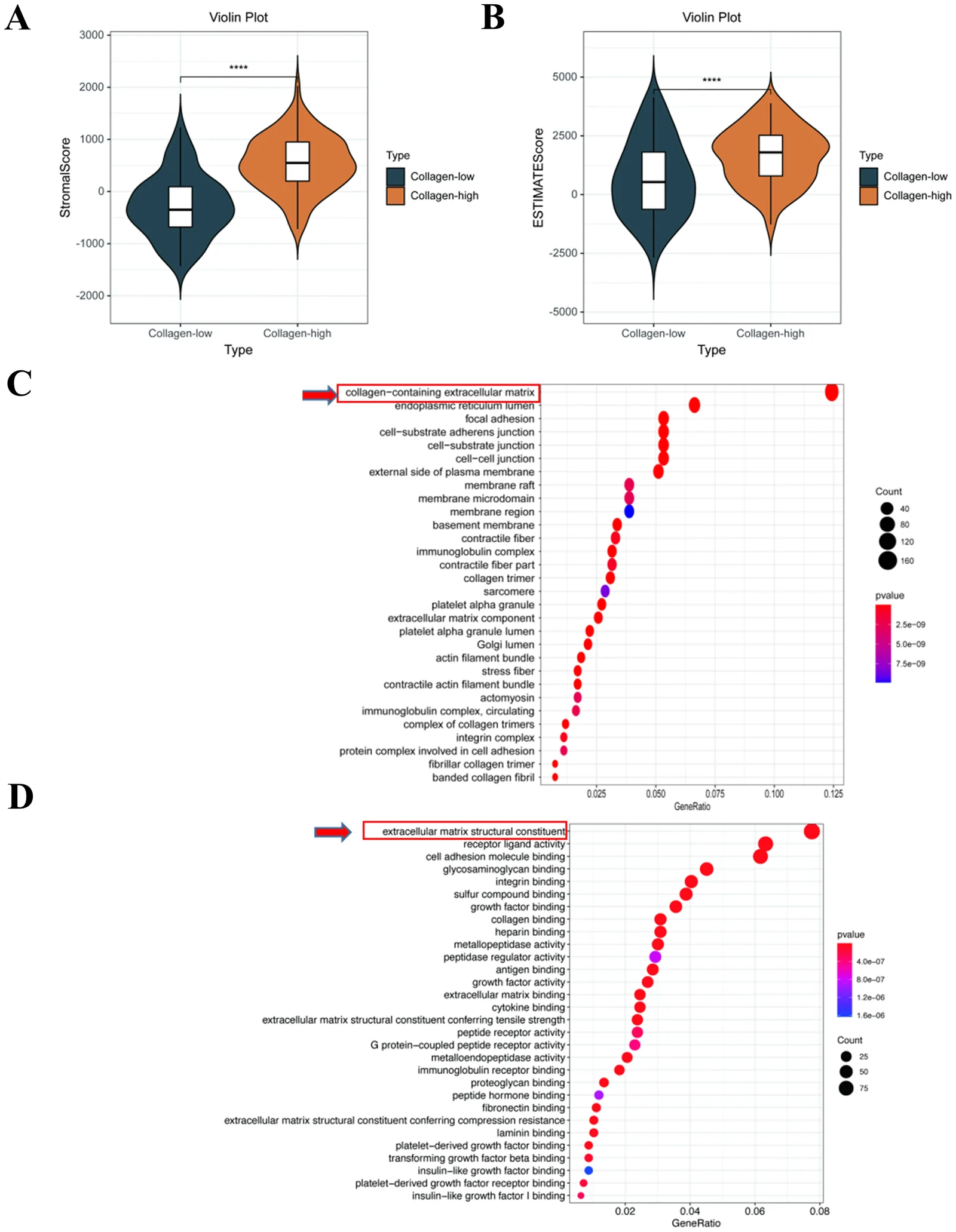
Collagen-related transcriptomic signatures are associated with increased stromal features in breast cancer. **(A, B)** Tumors were classified into collagen-high and collagen-low groups according to the median ssGSEA score of the REACTOME_COLLAGEN_FORMATION gene set. **(C, D)** Gene Ontology cellular-component and molecular-function enrichment analyses of collagen-associated genes. TCGA-derived results represent transcriptomic signatures and computational scores rather than direct pathological measurement of collagen deposition. Differences between collagen-high and collagen-low tumors were evaluated using the two-sided Wilcoxon rank-sum test. ****P < 0.0001.

To characterize collagen-associated stromal features in breast cancer, TCGA-BRCA tumors were stratified into collagen-high and collagen-low groups according to the transcriptome-derived collagen-formation score. Collagen-high tumors displayed significantly higher StromalScore and ESTIMATEScore values than collagen-low tumors (**Figures 1A, B**). Gene Ontology enrichment analysis of collagen-associated genes identified terms related to collagen-containing extracellular matrix, extracellular-matrix structural constituents, cell adhesion, and integrin binding (**Figures 1C, D**). Because these analyses were based on transcriptomic signatures and computational scores, they indicate an association with stromal and ECM-related features rather than direct pathological measurement of collagen deposition.

### COL I reduces ACSL5 protein abundance in TNBC cells

**Figure 2:**
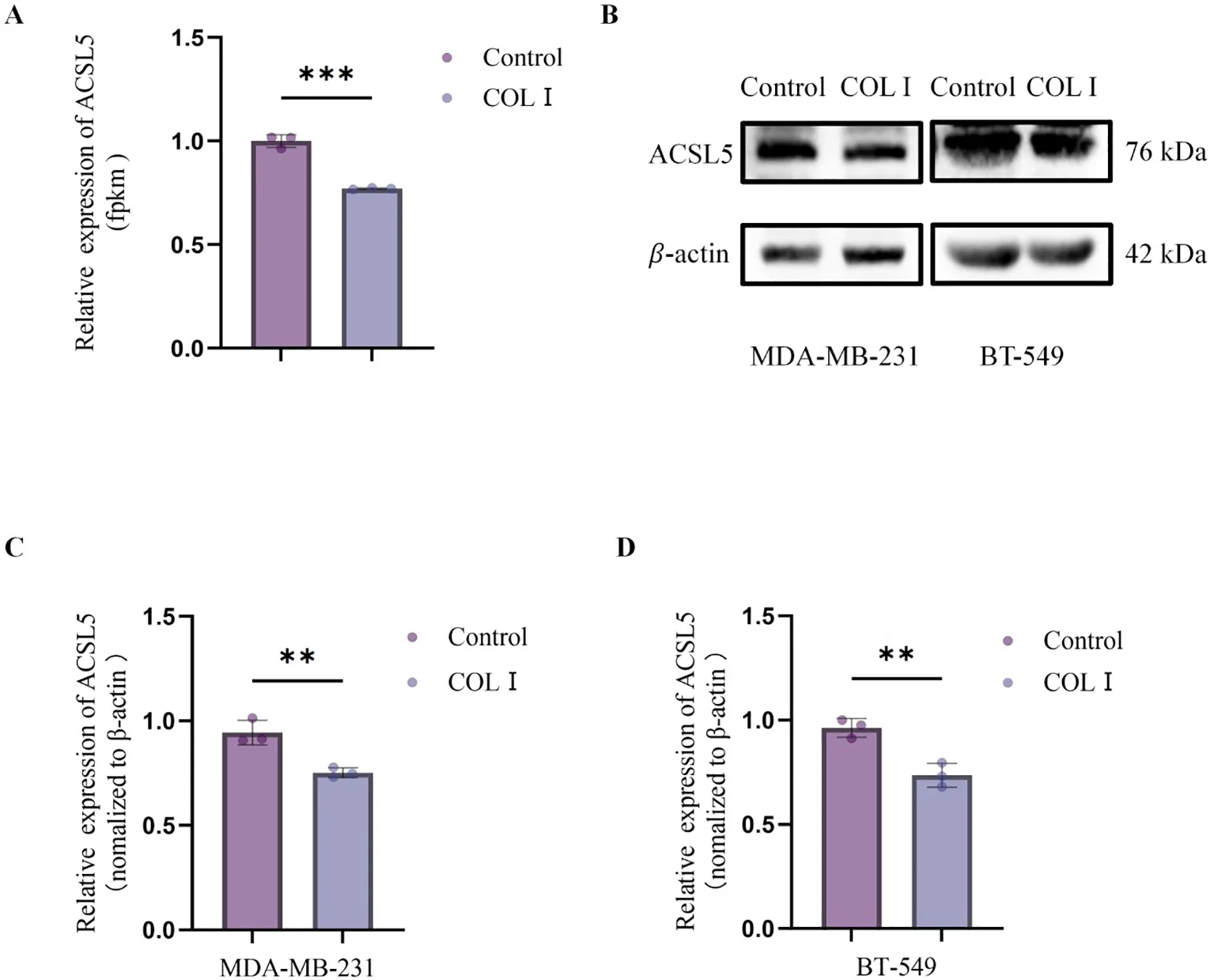
COL I reduces ACSL5 protein abundance in TNBC cells. **(A)** Transcriptome sequencing analysis of COL I-treated and control TNBC cells. **(B-D)** Western blotting and quantitative analysis of ACSL5 protein expression in MDA-MB-231 and BT-549 cells. Data are presented as mean ± SD from three independent biological experiments. Two-sided Student’s t-test; **P < 0.01, ***P < 0.001.

To explore downstream molecules regulated by COL I, we established COL I-treated TNBC cell models and performed transcriptome sequencing. The sequencing screen suggested a modest reduction in ACSL5 transcript abundance after COL I treatment (**Figure 2A**). Western blotting showed a more pronounced reduction in ACSL5 protein abundance in both MDA-MB-231 and BT-549 cells (**Figures 2B–D**). ACSL5 was carried forward for analysis of post-transcriptional regulation and protein stability.

### ACSL5 expression is associated with clinical and immune-infiltration features in public datasets

**Figure 3:**
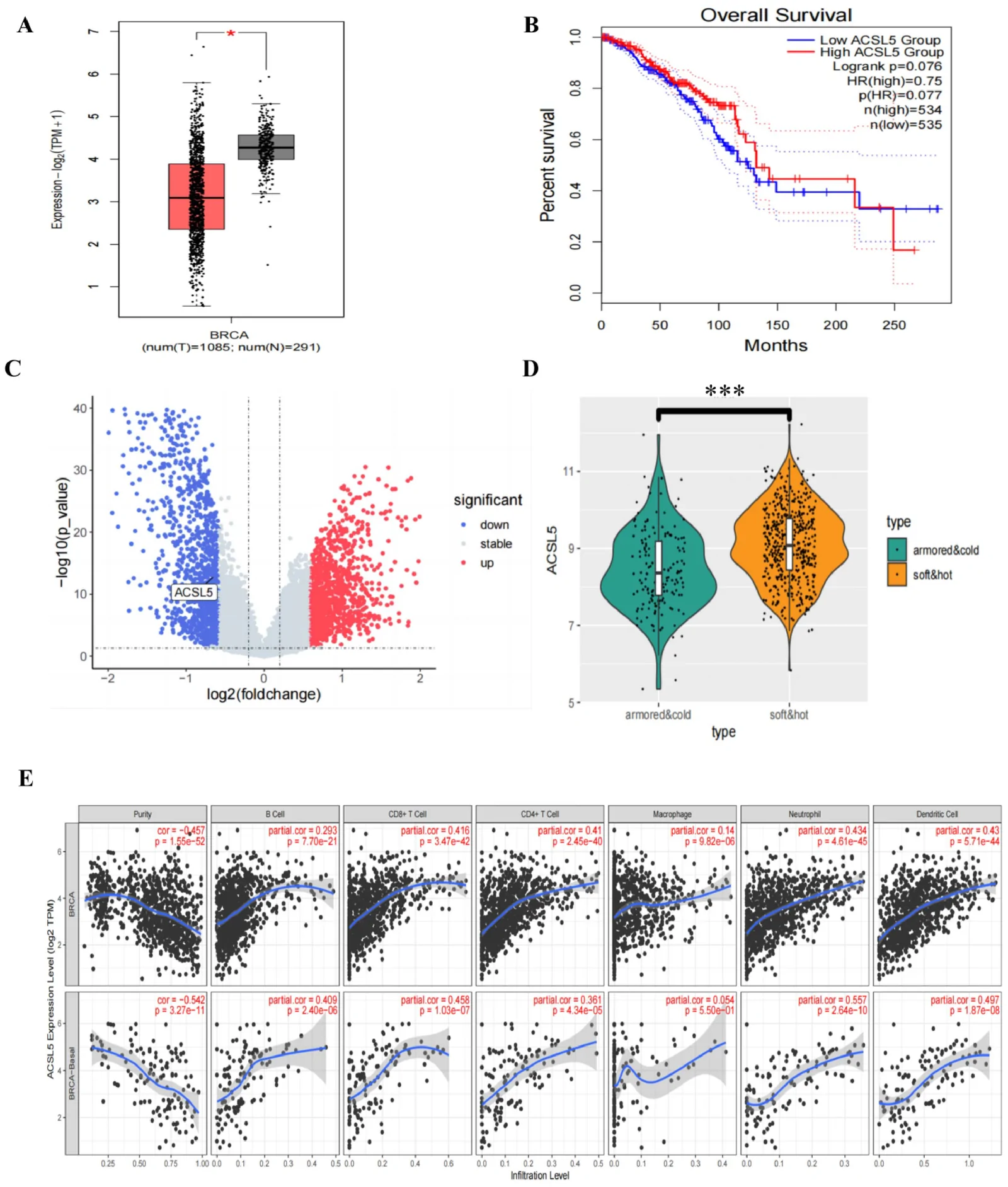
ACSL5 expression is associated with clinical and immune-infiltration features in public datasets. **(A)** GEPIA analysis of ACSL5 expression in breast cancer and normal tissues. **(B)** Kaplan–Meier overall-survival analysis according to ACSL5 expression. **(C)** Volcano plot of differential gene expression between the armored/cold and soft/hot TNBC subtypes, with ACSL5 highlighted. **(D)** Comparison of ACSL5 expression between the armored/cold and soft/hot subtypes. **(E)** TIMER analysis of the association between ACSL5 expression and estimated immune-cell infiltration in BRCA and basal-like BRCA. Statistical methods and sample sizes are shown in the corresponding source panels; ***P < 0.001 for **(D)**.

To explore the clinical and immune-related context of ACSL5, we analyzed GEPIA, TCGA-derived subtype data, and TIMER. ACSL5 expression was lower in breast cancer tissues than in normal tissues (**Figure 3A**). Higher ACSL5 expression showed a trend toward improved overall survival, but the difference did not reach statistical significance (log-rank P = 0.076; **Figure 3B**). Differential-expression and subtype analyses showed higher ACSL5 expression in the soft/hot subtype than in the armored/cold subtype (**Figures 3C, D**). TIMER analyses further showed positive associations between ACSL5 expression and estimated infiltration of several immune-cell populations in BRCA and basal-like BRCA (**Figure 3E**).

### COL I promotes malignant phenotypes in TNBC cells, whereas ACSL5 overexpression partially reverses these effects

**Figure 4:**
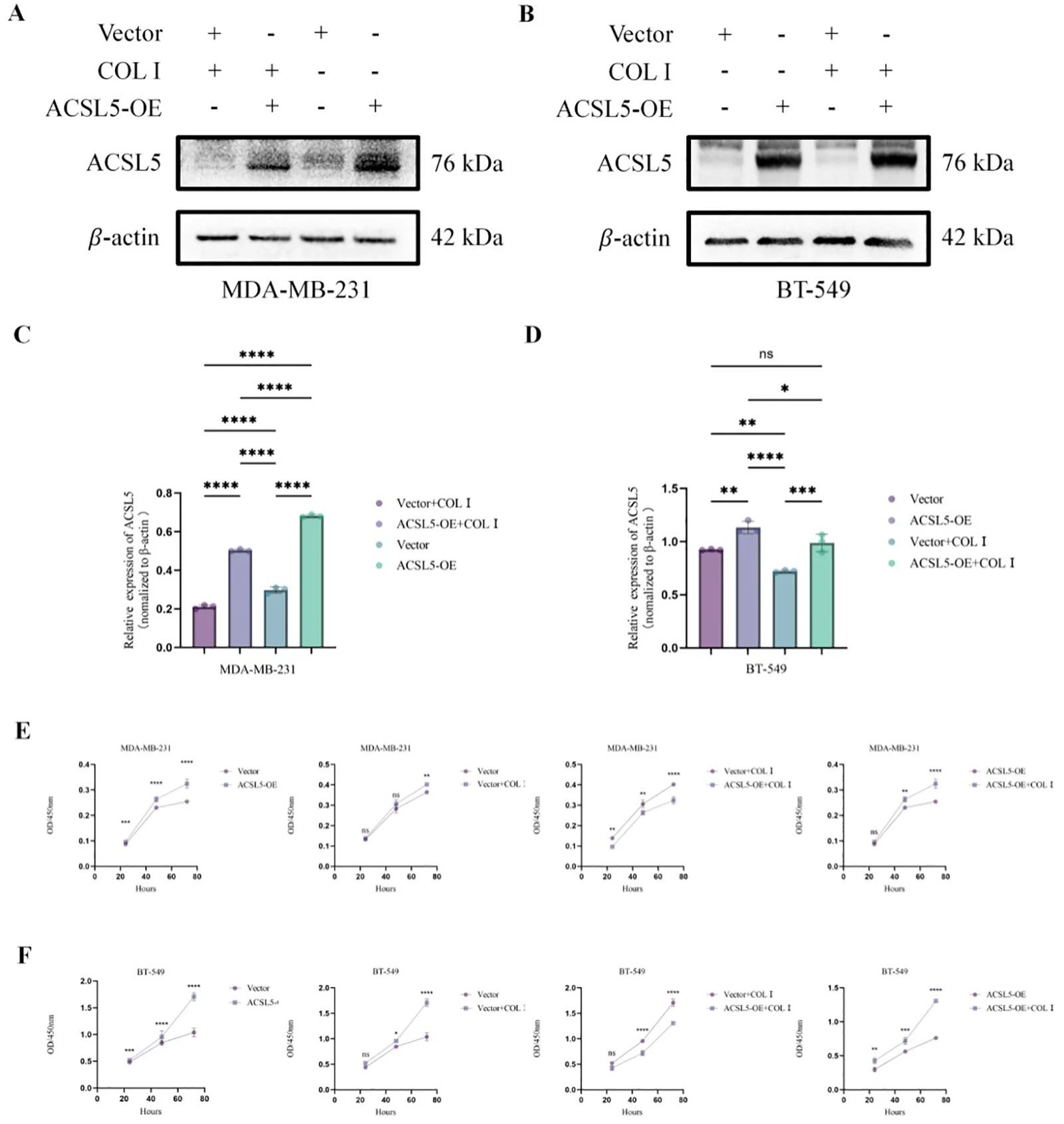
ACSL5 overexpression attenuates COL I-induced TNBC cell proliferation. **(A-D)** Western blotting and quantitative analysis of ACSL5 expression after COL I treatment and ACSL5 overexpression. **(E, F)** CCK-8 assay of cell proliferation at 24, 48, and 72 h. One-way ANOVA with Tukey’s test for western-blot quantification and two-way ANOVA with Tukey’s test for CCK-8 assays; ns, not significant; *P < 0.05, **P < 0.01, ***P < 0.001, ****P < 0.0001. Data are presented as mean ± SD from three independent biological experiments.

**Figure 5:**
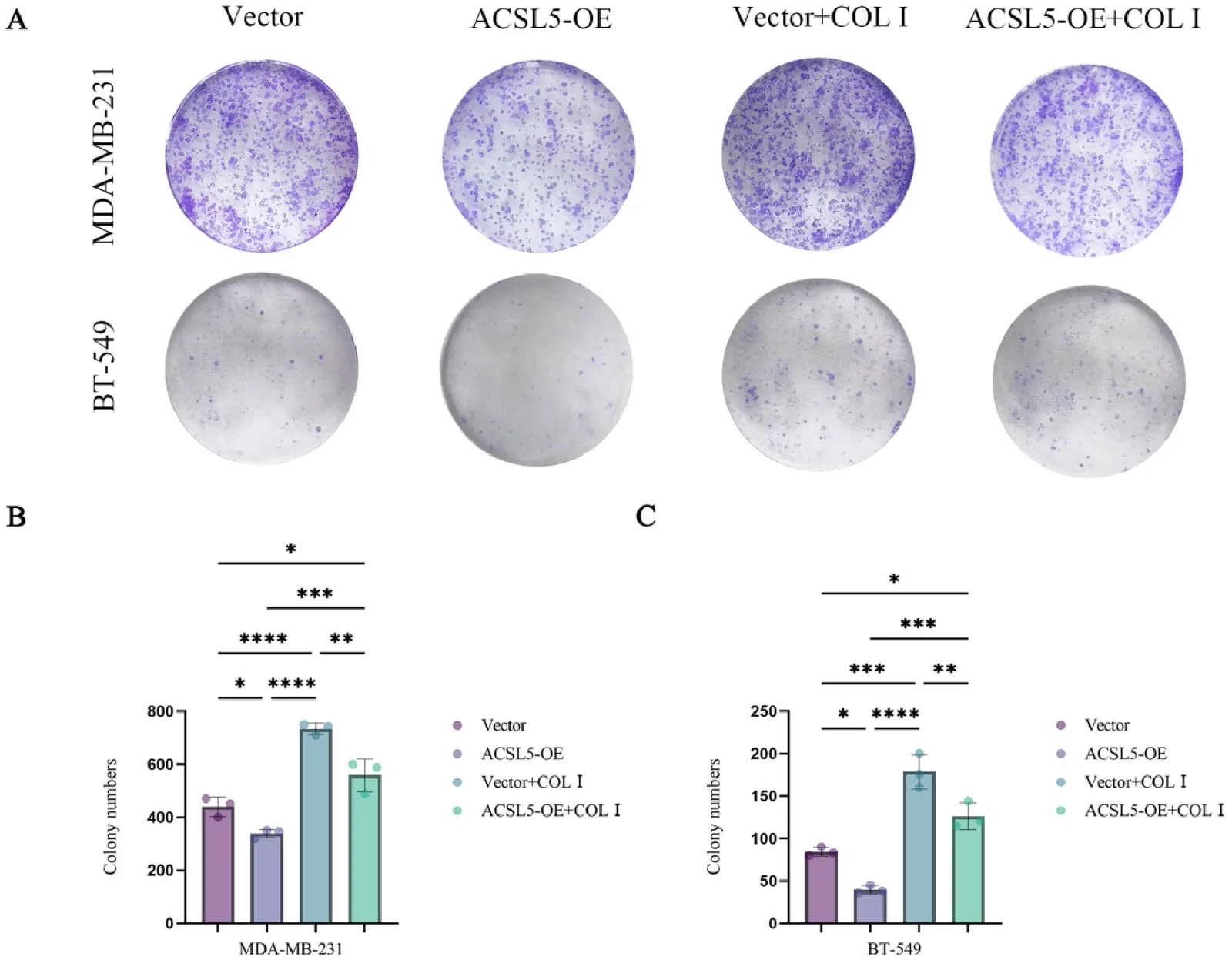
ACSL5 overexpression attenuates COL I-induced colony formation. **(A)** Representative colony-formation images from MDA-MB-231 and BT-549 cells assigned to Vector, ACSL5-OE, Vector + COL I, and ACSL5-OE + COL I groups. **(B, C)** Quantification of colony numbers. Data are presented as mean ± SD from three independent biological experiments. One-way ANOVA followed by Tukey’s multiple-comparison test; *P < 0.05, **P < 0.01, ***P < 0.001, ****P < 0.0001.

To examine the functional relationship between COL I and ACSL5, ACSL5 was overexpressed in the presence or absence of COL I. Western blotting confirmed ACSL5 overexpression and showed that enforced ACSL5 expression partially restored ACSL5 abundance under COL I treatment (**Figures 4A–D**). CCK-8 assays showed that COL I increased TNBC cell proliferation, whereas ACSL5 overexpression reduced proliferation and partially attenuated the COL I-induced increase (**Figures 4E, F**). Colony-formation assays similarly showed that COL I enhanced long-term proliferative capacity, whereas ACSL5 overexpression reduced colony formation and weakened the effect of COL I (**Figures 5A–C**).

**Figure 6:**
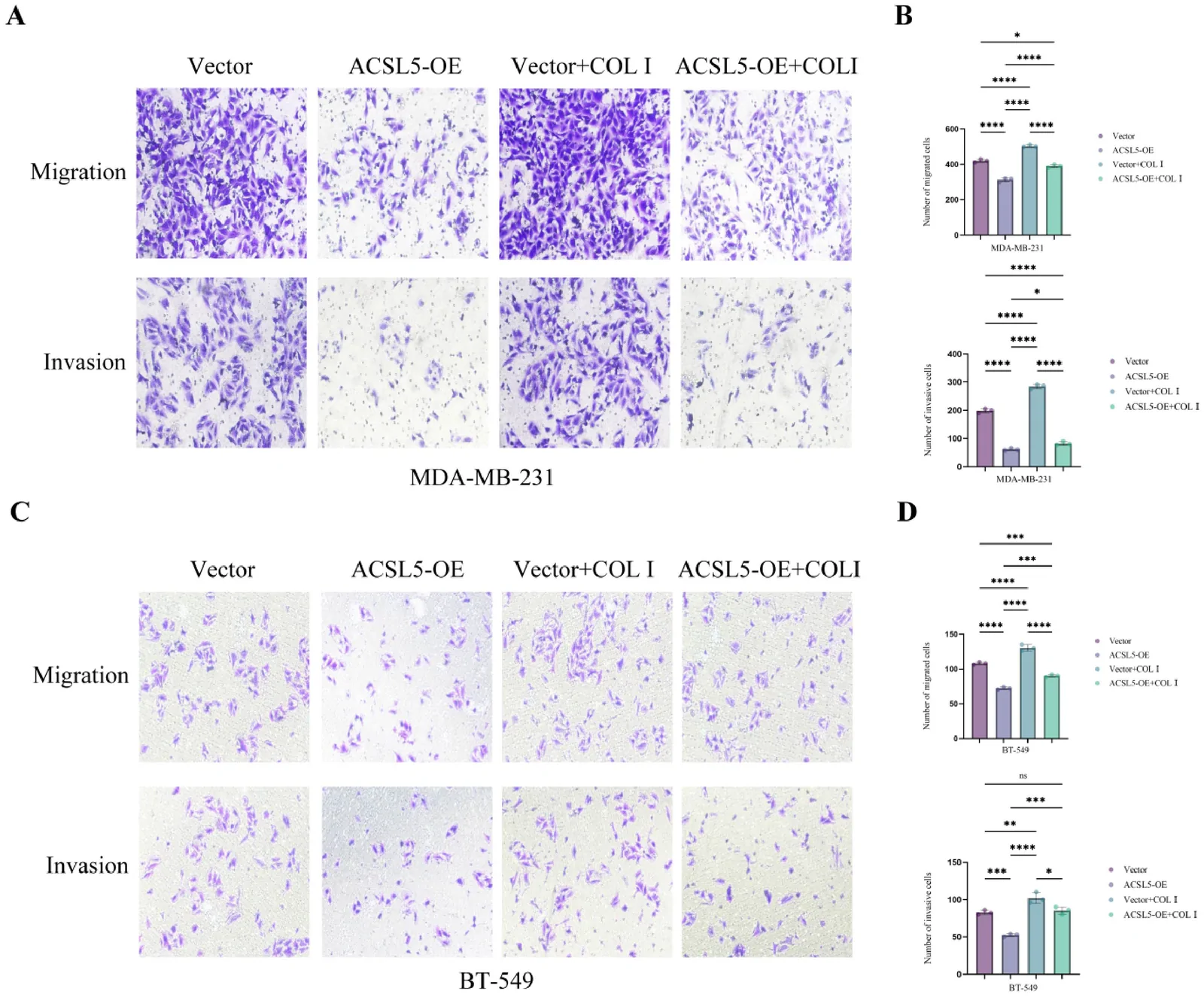
ACSL5 overexpression attenuates COL I-induced migration and invasion. **(A, B)** Representative Transwell images and quantification of migration and invasion in MDA-MB-231 cells. **(C, D)** Representative images and quantification in BT-549 cells. Cells were assigned to Vector, ACSL5-OE, Vector + COL I, and ACSL5-OE + COL I groups. Data are presented as mean ± SD from three independent biological experiments. One-way ANOVA followed by Tukey’s multiple-comparison test; ns, not significant; *P < 0.05, **P < 0.01, ***P < 0.001, ****P < 0.0001.

Transwell assays showed that COL I increased migration and invasion in MDA-MB-231 and BT-549 cells, whereas ACSL5 overexpression suppressed these phenotypes. Compared with COL I treatment alone, ACSL5 overexpression in COL I-treated cells significantly reduced migration and invasion (**Figures 6A–D**). Together, these findings support ACSL5 as a downstream functional component that restrains COL I-induced malignant behavior in TNBC cells.

### COL I is associated with accelerated ACSL5 protein loss

**Figure 7:**
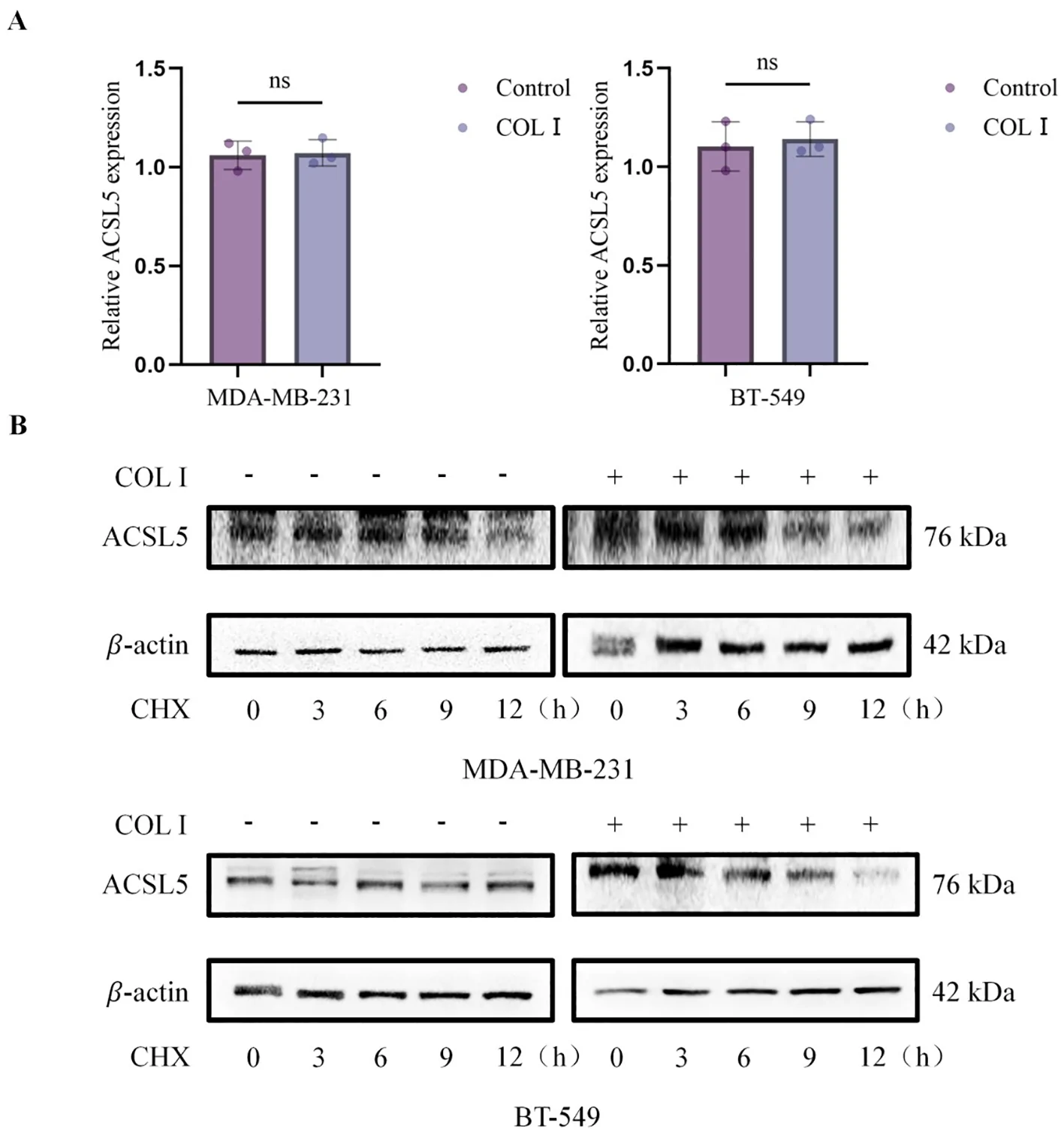
COL I is associated with accelerated ACSL5 protein loss. **(A)** qPCR analysis of ACSL5 mRNA expression following COL I treatment in MDA-MB-231 and BT-549 cells. **(B)** Representative CHX-chase western blots showing ACSL5 protein abundance at 0, 3, 6, 9, and 12 h after CHX treatment in control and COL I-treated MDA-MB-231 and BT-549 cells. ACSL5 mRNA data are presented as mean ± SD from three independent biological experiments. Two-sided Student’s t-test was used for **(A)** ns, not significant.

Although COL I reduced ACSL5 protein abundance, qPCR showed no significant change in ACSL5 mRNA expression in either MDA-MB-231 or BT-549 cells (**Figure 7A**), suggesting that the regulation predominantly occurs at the post-transcriptional level. We next performed CHX-chase experiments to assess ACSL5 protein stability. Representative western blots showed a more rapid decrease in ACSL5 protein abundance over time in COL I-treated cells than in untreated controls in both TNBC cell lines (**Figure 7B**). These findings are consistent with accelerated ACSL5 protein loss following COL I treatment.

### COL I-induced ACSL5 loss is sensitive to proteasome inhibition

**Figure 8:**
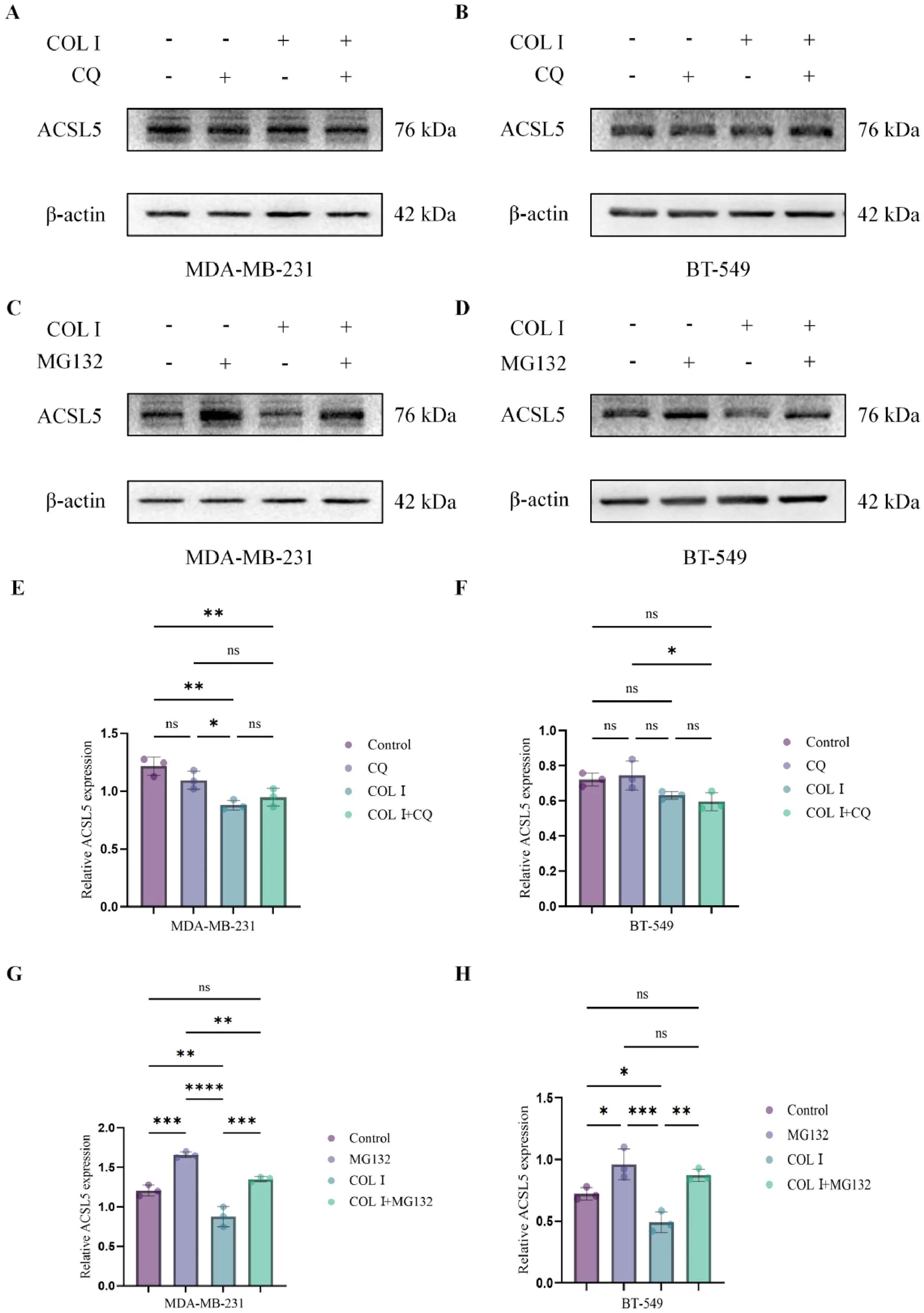
Proteasome inhibition restores ACSL5 after COL I treatment. **(A, B)** Representative western blots of ACSL5 after CQ treatment in MDA-MB-231 and BT-549 cells. **(C, D)** Representative western blots after MG132 treatment. **(E, F)** Quantification of CQ experiments. **(G, H)** Quantification of MG132 experiments. Data are presented as mean ± SD from three independent biological experiments. One-way ANOVA followed by Tukey’s multiple-comparison test; ns, not significant; *P < 0.05, **P < 0.01, ***P < 0.001, ****P < 0.0001.

To determine the pathway involved in COL I-induced ACSL5 loss, we inhibited the autophagy–lysosome pathway with CQ and the proteasome with MG132. CQ did not consistently restore ACSL5 after COL I treatment (**Figures 8A, B, E, F**), whereas MG132 substantially increased ACSL5 abundance and reversed the COL I-induced reduction in both cell lines (**Figures 8C, D, G, H**). These results support a predominant contribution of proteasome-sensitive degradation.

### COL I upregulates UBE2V1 and modulates its association with ACSL5

**Figure 9:**
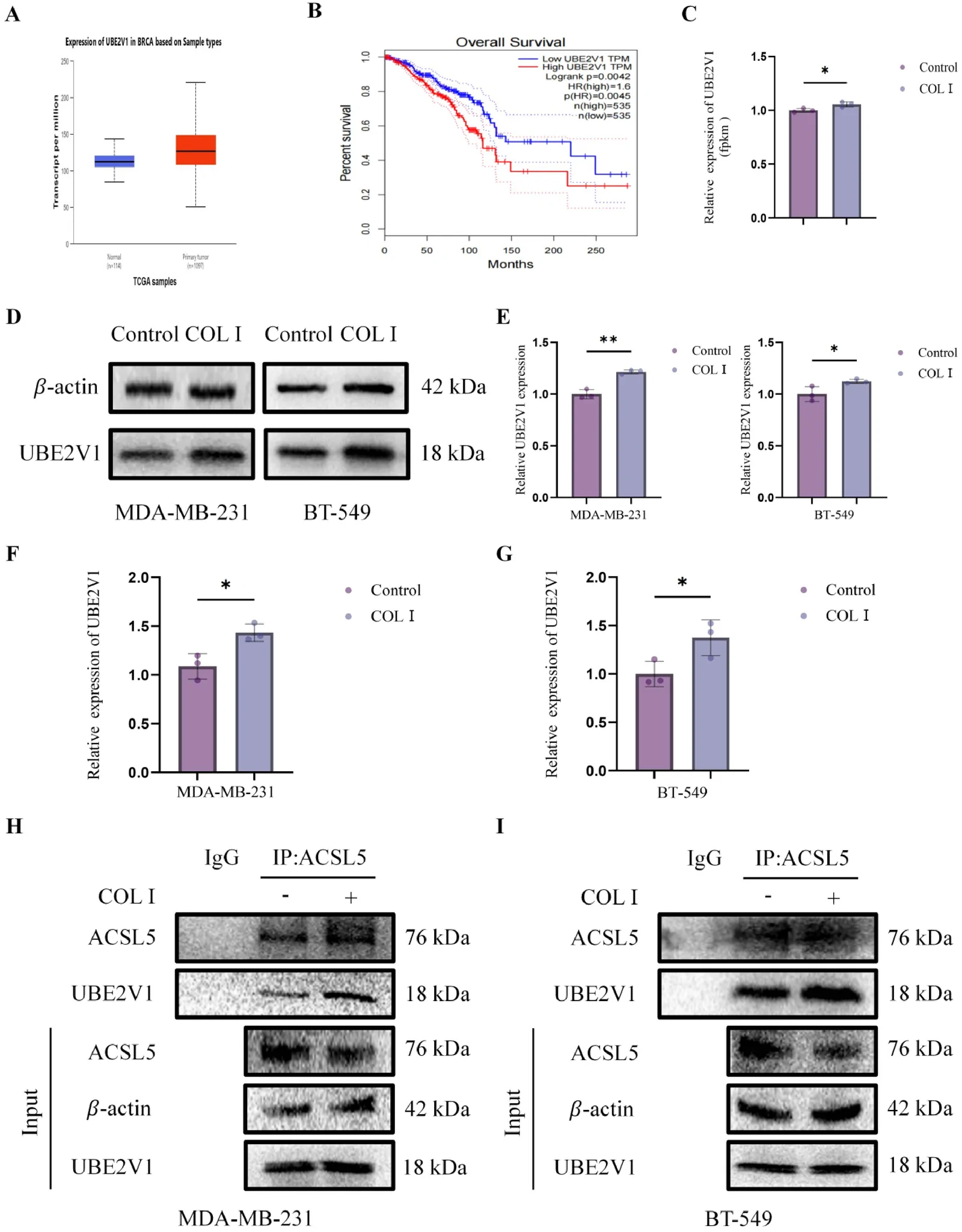
COL I upregulates UBE2V1 and modulates its association with ACSL5. **(A)** UBE2V1 expression in breast tumors and normal tissues in TCGA/UALCAN. **(B)** Kaplan–Meier overall-survival analysis according to UBE2V1 expression. **(C)** UBE2V1 expression in transcriptome-sequencing data following COL I treatment. **(D, E)** Western-blot analysis and corresponding densitometric quantification of UBE2V1 protein abundance in MDA-MB-231 and BT-549 cells. **(F, G)** qPCR analysis of UBE2V1 expression following COL I treatment. **(H, I)** Representative ACSL5 co-immunoprecipitation assays demonstrating the association between ACSL5 and UBE2V1 in MDA-MB-231 and BT-549 cells. Representative Co-IP blots showed an apparently stronger UBE2V1 signal following COL I treatment. Data are presented as mean ± SD from three independent biological experiments where quantitative analyses are shown. Two-sided Student’s t-test; *P < 0.05, **P < 0.01.

To identify candidate regulators associated with ACSL5 protein stability, ACSL5-associated proteins were screened by IP–mass spectrometry. UBE2V1 was prioritized by integrating the mass-spectrometry results with protein-homeostasis annotations, transcriptome data, and public-dataset evidence. UBE2V1 expression was higher in breast tumors than in normal tissues, and higher UBE2V1 expression was associated with poorer overall survival (**Figures 9A, B**). Transcriptome, qPCR, and western blot analyses showed that COL I increased UBE2V1 expression in TNBC cells (**Figures 9C–G**). Co-immunoprecipitation confirmed an association between UBE2V1 and ACSL5 in both TNBC cell lines. In the representative blots, the UBE2V1 signal co-immunoprecipitated with ACSL5 appeared stronger following COL I treatment (**Figures 9H, I**), suggesting that COL I may enhance the association between UBE2V1 and ACSL5.

### COL I treatment is associated with stronger total ACSL5 ubiquitination signals

**Figure 10:**
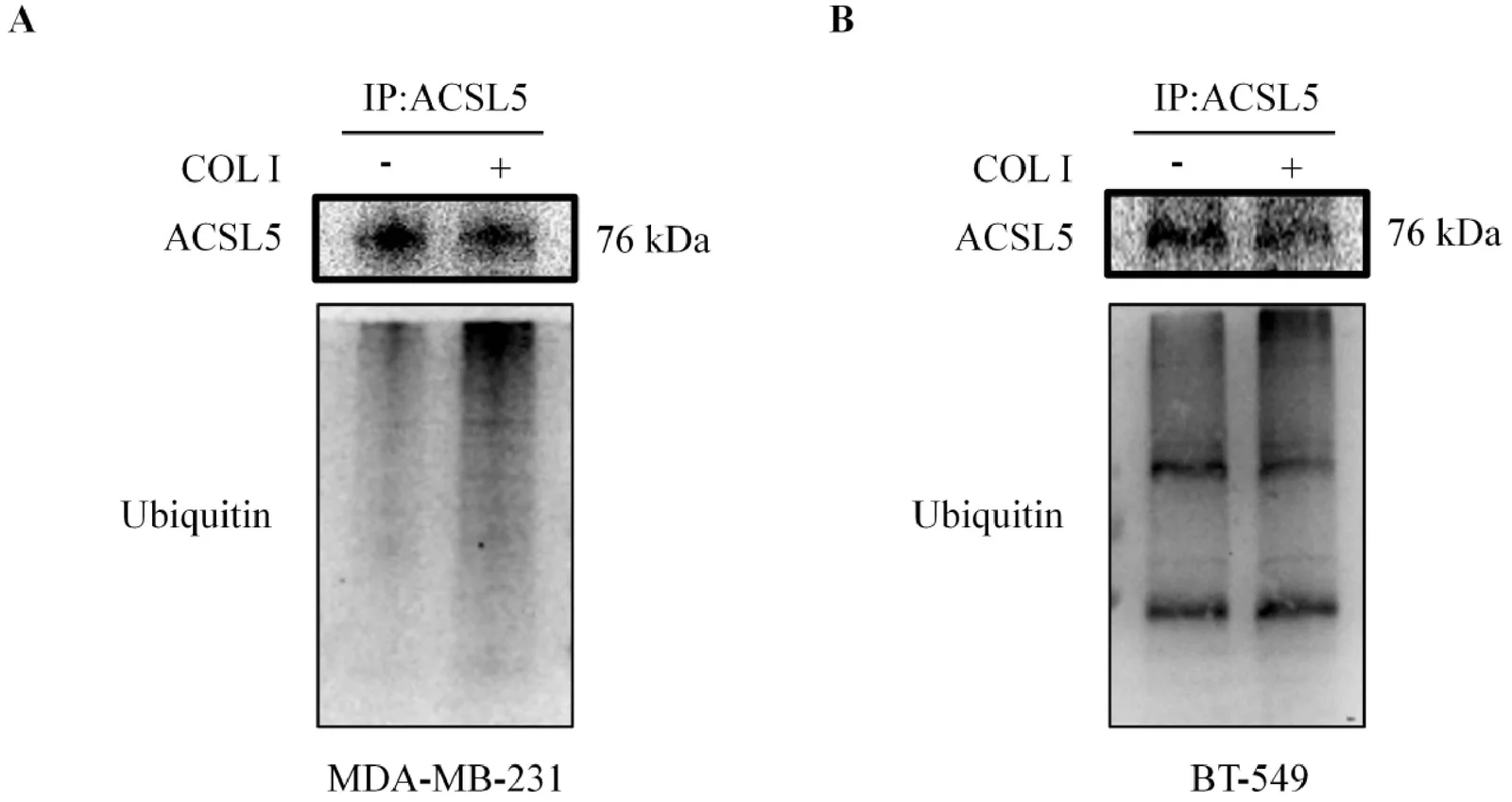
COL I treatment is associated with stronger total ACSL5 ubiquitination signals. **(A, B)** Representative ACSL5 immunoprecipitation followed by pan-ubiquitin western blotting in MDA-MB-231 and BT-549 cells following MG132 pretreatment.

To examine whether COL I treatment is associated with altered ACSL5 ubiquitination, cells were pretreated with MG132, ACSL5 was immunoprecipitated, and ubiquitin signals were detected using a pan-ubiquitin antibody. Representative ubiquitination assays showed stronger high-molecular-weight ubiquitin signals associated with immunoprecipitated ACSL5 following COL I treatment in both MDA-MB-231 and BT-549 cells (**Figures 10A, B**). Together with the MG132 rescue experiments, these findings are consistent with an association between enhanced ACSL5 ubiquitination and proteasome-sensitive ACSL5 loss.

### UBE2V1 knockdown partially restores ACSL5 expression and attenuates COL I-induced migration and invasion

**Figure 11:**
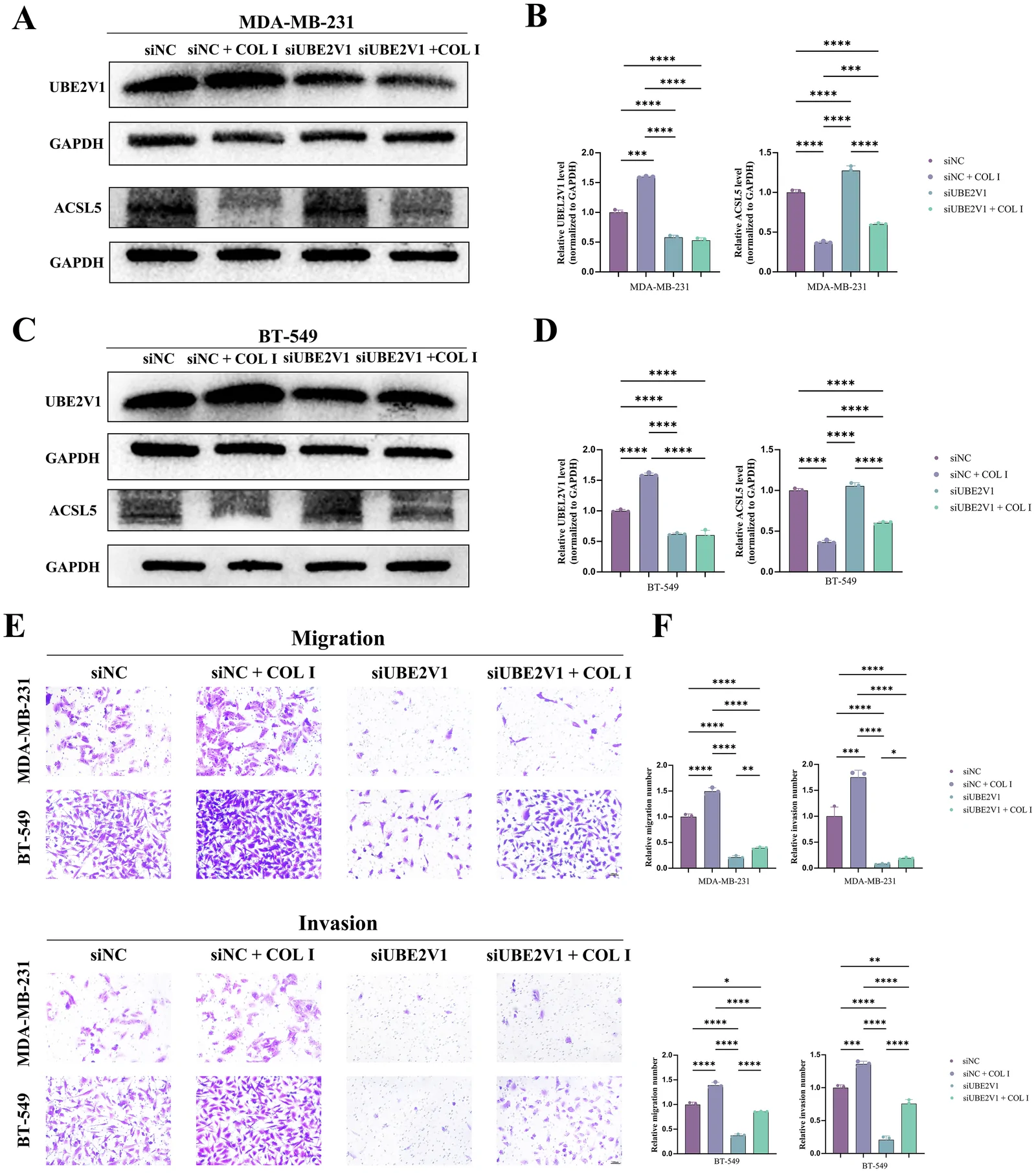
UBE2V1 knockdown partially restores ACSL5 protein expression and attenuates COL I-induced migration and invasion. **(A, B)** Representative western blots and corresponding densitometric analyses of UBE2V1 and ACSL5 in MDA-MB-231 cells assigned to siNC, siNC + COL I, siUBE2V1, and siUBE2V1 + COL I groups. **(C, D)** Corresponding western-blot and densitometric analyses in BT-549 cells. **(E, F)** Representative Transwell migration and invasion images and corresponding quantification in MDA-MB-231 and BT-549 cells. Data are presented as mean ± SD from three independent biological experiments. One-way ANOVA followed by Tukey’s multiple-comparison test; *P < 0.05, **P < 0.01, ***P < 0.001, ****P < 0.0001.

To determine whether UBE2V1 is functionally required for COL I-induced ACSL5 downregulation, UBE2V1 was depleted using siRNA in MDA-MB-231 and BT-549 cells. Western blotting confirmed efficient UBE2V1 knockdown in both cell lines. In siNC-transfected cells, COL I increased UBE2V1 expression and reduced ACSL5 protein abundance. Importantly, UBE2V1 knockdown partially restored ACSL5 protein expression in the presence of COL I in both cell lines (**Figures 11A–D**).

We next examined whether UBE2V1 contributes to COL I-induced migration and invasion. COL I significantly increased both migration and invasion in MDA-MB-231 and BT-549 cells, whereas UBE2V1 knockdown markedly attenuated these effects (**Figures 11E, F**). These findings provide loss-of-function evidence that UBE2V1 is required, at least in part, for COL I-induced ACSL5 downregulation and malignant cell behavior.

## Discussion

TNBC progression is shaped by reciprocal signaling between tumor cells and the surrounding ECM (12, 17). In this study, collagen-related transcriptomic signatures were associated with higher stromal and ESTIMATE scores, and exogenous COL I promoted proliferation, colony formation, migration, and invasion in TNBC cells. Mechanistically, COL I reduced ACSL5 protein abundance without significantly changing ACSL5 mRNA, accelerated ACSL5 protein loss, and increased total ACSL5 ubiquitination. IP–mass spectrometry and co-immunoprecipitation identified UBE2V1 as an ACSL5-associated regulatory component whose expression and association with ACSL5 were enhanced by COL I. Most importantly, UBE2V1 knockdown partially restored ACSL5 protein expression and significantly attenuated COL I-induced migration and invasion in both TNBC cell lines, providing loss-of-function evidence that UBE2V1 is functionally required, at least in part, for these COL I-induced tumor-cell phenotypes.

ACSL5 is involved in long-chain fatty-acid activation and has context-dependent roles in cancer (30, 31). Previous work has also linked ACSL5 to antigen presentation and cancer immunity (22). In our study, ACSL5 was lower in breast tumors than in normal tissues, and higher ACSL5 showed a nonsignificant trend toward better overall survival. ACSL5 was also higher in the soft/hot than in the armored/cold TNBC subtype and correlated with estimated immune-cell infiltration in public datasets. These analyses provide biological context but remain associative. Our direct functional data are restricted to tumor-cell proliferation, colony formation, migration, and invasion. The relationship between collagen-rich and immune-excluded tumor phenotypes is likely multifactorial and should not be attributed solely to ACSL5 regulation. In addition to potential tumor-cell-intrinsic changes, dense collagen networks may physically restrict immune-cell trafficking, while increased matrix stiffness and collagen-receptor signaling can alter mechanotransduction and stromal–tumor communication. Stromal-cell-derived factors and additional tumor-cell-intrinsic immunoregulatory pathways may also independently influence immune-cell recruitment and function. Therefore, ACSL5 downregulation should be regarded as one potential component associated with a collagen-rich tumor state rather than a complete causal explanation for immune exclusion.

The protein-stability experiments support a proteasome-sensitive mechanism for COL I-induced ACSL5 loss. Representative CHX-chase blots were consistent with accelerated ACSL5 protein loss following COL I treatment, whereas MG132 partially restored ACSL5 abundance. In addition, representative ubiquitination assays showed stronger total ubiquitin signals associated with ACSL5 following COL I treatment. Together, these observations support an association between COL I exposure, enhanced ACSL5 ubiquitination, and proteasome-sensitive ACSL5 loss. UBE2V1 knockdown further demonstrated that UBE2V1 is functionally required for ACSL5 downregulation under COL I exposure. Nevertheless, these findings do not show that UBE2V1 directly catalyzes ubiquitin transfer to ACSL5. UBE2V1 is a catalytically inactive E2 variant that usually functions with a catalytic partner such as UBE2N/UBC13 (23–25). The relevant catalytic E2, E3 ligase, ubiquitin-chain linkage, and ACSL5 acceptor lysine residues were not defined. Accordingly, the present data support UBE2V1-associated regulation of ACSL5 stability rather than a fully resolved direct ubiquitination reaction.

From a translational perspective, targeting a downstream regulatory component may be more feasible than directly disrupting COL I, which is broadly required for tissue structure and repair (13, 32, 33). The ability of UBE2V1 knockdown to partially restore ACSL5 expression and suppress COL I-induced migration and invasion identifies the UBE2V1–ACSL5 relationship as a potential regulatory vulnerability that warrants further investigation. However, the current evidence is limited to cell-based models, and neither therapeutic efficacy nor selectivity has been established. Any translational interpretation should therefore remain provisional until the pathway is validated in genetically controlled animal models and clinical specimens.

This study has several limitations. First, the current evidence is derived mainly from public datasets and *in vitro* cell-line experiments, and the COL I–UBE2V1–ACSL5 relationship has not been validated *in vivo*. Second, although UBE2V1 knockdown partially restored ACSL5 expression and attenuated COL I-induced migration and invasion in both TNBC cell lines, the study did not directly test whether UBE2V1 depletion reduces ACSL5 ubiquitination. The catalytic E2 partner, relevant E3 ligase, ubiquitin-chain topology, and ACSL5 ubiquitination sites therefore remain unresolved. Third, the receptor and intracellular signaling pathway through which extracellular COL I increases UBE2V1 expression were not examined. Fourth, the immune-related findings were obtained from public-dataset correlations and were not supported by direct antigen-presentation or T-cell functional assays. Finally, the expression relationships among collagen-related features, UBE2V1, and ACSL5 require validation in an independent clinical tissue cohort. These limitations define important directions for future biochemical, *in vivo*, immune-functional, and clinical studies.

## Conclusion

This study identifies a COL I–UBE2V1–ACSL5 regulatory relationship that contributes to malignant phenotypes in TNBC cells. COL I reduces ACSL5 protein abundance through a proteasome-sensitive process and is associated with stronger total ACSL5 ubiquitination signals, increased UBE2V1 expression, and enhanced UBE2V1–ACSL5 association. UBE2V1 knockdown partially restores ACSL5 protein expression and attenuates COL I-induced migration and invasion in both TNBC cell lines, indicating that UBE2V1 is functionally required, at least in part, for this regulatory phenotype. Further studies are required to define the complete ubiquitination machinery and determine the *in vivo*, clinical, and immune relevance of this relationship.

## Data Availability

The original contributions presented in the study are included in the article/Supplementary Material. Further inquiries can be directed to the corresponding authors.
